# Projection-Free CLIP-Scale EEG Latents via a U-Net-Style Autoencoder

**DOI:** 10.3390/s26144583

**Published:** 2026-07-20

**Authors:** Jeyoung Lee, Jaekwan Ahn, Jaeseung Sim, Hochul Kang

**Affiliations:** 1 School of Computer Science and Engineering, Soongsil University, 369 Sangdo-ro, Dongjak-gu, Seoul 06978, Republic of Korea; dlwpdud@ssu.ac.kr; 2 Sweetndata Inc., Seoul 06224, Republic of Korea; jakey.ahn@sweetndata.com (J.A.); sjs@sweetndata.com (J.S.); 3 Department of Digital Media Engineering, The Catholic University of Korea, Bucheon-si 14662, Republic of Korea

**Keywords:** electroencephalography (EEG), generative AI, representation learning, U-Net, cross-modal alignment, signal reconstruction

## Abstract

Electroencephalography is emerging as a promising conditioning modality for generative visual models. However, existing representation learning approaches often rely on high-capacity masked autoencoders and complex projection networks. When constrained to compact embedding dimensions to match vision-language models, these heavy transformer-based bottlenecks frequently suffer from representation collapse and lose critical signal dynamics. To address this, we propose a lightweight and projection-free autoencoder that directly outputs compact, Contrastive Language–Image Pre-training (CLIP)-scale latent vectors trained toward the CLIP embedding space. Our model adopts a U-Net-style architecture combining one-dimensional convolutional residual blocks for temporal dynamics and inter-channel attention modules for spatial dependencies, alongside skip connections to ensure stable reconstruction. Extensive experiments on visual perception datasets demonstrate that our approach successfully tracks complex signal amplitudes without collapsing. Under strict dimensional constraints, the proposed model achieves superior signal reconstruction fidelity across time and frequency domains using significantly fewer parameters than traditional masked autoencoder baselines. Furthermore, latent space visualizations and zero-shot retrieval tasks reveal that while the baseline collapses toward unstructured, near-chance representations, our architecture preserves emerging, partial semantic organization and retrieves several times above chance. This indicates that the proposed design preserves signal structure while exhibiting preliminary, above-chance semantic alignment, enabling integration into brain-driven generative pipelines.

## 1. Introduction

Recent advances in generative visual models, particularly diffusion-based approaches [1,2], have expanded the range of modalities used as conditioning signals. While text and images remain the most widely adopted forms of control, leveraging neurophysiological signals as alternative conditioning modalities has gained traction to capture implicit cognitive and perceptual states. Among these signals, electroencephalography provides a non-invasive and temporally rich measurement of brain activity and has recently been explored for visual decoding tasks. As a wearable physiological sensing modality, EEG also poses a representation problem of independent interest: extracting robust, compact representations from noisy multi-channel sensor signals under severe dimensional compression, which is the focus of this work.

Most existing brain-driven image generation studies adopt latent diffusion models conditioned on pretrained multimodal encoders such as Contrastive Language–Image Pre-training (CLIP) [3]. In this paradigm, the structural quality of the conditioning representation determines the effectiveness of the generative process. However, contemporary representation learning frameworks frequently rely on high-capacity masked autoencoders, such as Ti-MAE [4], to process the signals. Because these transformer-dominant architectures do not naturally output compact representations matching CLIP embedding spaces, additional projection networks must be introduced. This increases system complexity and introduces confounding factors unrelated to the intrinsic quality of the learned representations.

Furthermore, applying large transformer-based architectures to brainwave data presents practical limitations. These datasets are typically limited in size and exhibit substantial inter-subject variability alongside strong temporal dynamics. Under these conditions, heavily parameterized models tend to suffer severe performance degradation when forced into a compact bottleneck. Specifically, when the latent space is constrained to match the dimensionality of CLIP embeddings, these models frequently experience representation collapse, resulting in an inability to track complex signal amplitudes and degenerating into unorganized noise clusters.

To address these challenges, we propose a lightweight and projection-free autoencoder that directly produces compact latent representations aligned with CLIP dimensions. Rather than adapting masked autoencoder architectures, we design a U-Net [5]-style framework explicitly tailored to the structural properties of time-series data. The proposed architecture combines one-dimensional convolutional residual blocks to capture local temporal patterns with inter-channel attention modules to model global spatial relationships. Skip connections between the encoder and decoder further ensure stable signal reconstruction while preserving fine-grained temporal information.

Crucially, unlike prior works that primarily evaluate representation quality through downstream image synthesis, we assess the encoder directly without relying on downstream generative synthesis. Downstream generation heavily relies on the powerful generative priors of diffusion models, which can obscure the true quality of the underlying signal embeddings. Instead, we directly measure signal reconstruction fidelity using established time and frequency domain metrics, including Mean Squared Error, Mean Absolute Error, Pearson Correlation Coefficient, and Fast Fourier Transform Cosine Similarity. We also validate cross-modal semantic alignment through zero-shot retrieval tasks and latent space clustering.

## 2. Related Work

### 2.1. EEG-Based Representation Learning

Representation learning for electroencephalography signals has been extensively studied, with autoencoder-based approaches serving as a widely adopted foundation for extracting compact and transferable features. Beyond simple reconstruction, recent research has actively explored self-supervised objectives, contrastive learning, and transformer-based masked modeling to improve generalization across subjects and tasks under limited label conditions.

Traditional self-supervised learning for brainwave representation has evolved through various neural architectures. Early methodologies explored general feature extraction paradigms [6], distributed concept representations [7], and denoising autoencoders [8]. Subsequent research integrated convolutional and recurrent networks to capture spatio-temporal dynamics [9] and utilized multi-task autoencoders to achieve robust classification performance [10]. More recently, the focus has shifted toward advanced unsupervised techniques, including contrastive learning paradigms [11,12], variational autoencoders for affective and medical classification [13,14], and temporal autoencoders for semi-supervised clustering [15,16].

Notably, transformer-based masked autoencoders have gained significant traction. Researchers have adapted masked autoencoders for general signal representation [17], polysomnography reconstruction [18], and topology-agnostic large-scale pre-training [19]. However, while these studies establish a robust foundation for signal encoding, few are explicitly designed to produce latent vectors directly compatible with the semantic embedding spaces used in modern vision-language models like CLIP [3]. This dimensional and semantic gap motivates the architectural focus of our proposed projection-free approach.

More recently, a line of general-purpose EEG foundation models has advanced rapidly, including LaBraM [20], EEGPT [21], CBraMod [22], and EEGFormer [23], which learn broad EEG representations through large-scale self-supervised pre-training across heterogeneous electrode montages and tasks. We acknowledge this line of work explicitly. However, the present study is scoped not as a general-purpose EEG foundation model but as the backbone of a CLIP-conditioned generative pipeline for reconstructing perceived images from EEG. Within this specific setting, the de facto standard backbone is the time-series masked autoencoder Ti-MAE [4], as adopted by recent EEG-to-image generative pipelines, which is why we select Ti-MAE as our baseline rather than the broad foundation models above: those target a different problem setting, namely general-purpose representation learning across diverse montages and tasks, rather than the CLIP-conditioned generative backbone studied here. We further emphasize that these general-purpose foundation models are trained to produce task-agnostic representations transferable across heterogeneous electrode montages and downstream tasks, and they do not natively emit a CLIP-scale 768-dimensional latent suited to a CLIP-conditioned generative backbone; adapting them to our setting would require appending exactly the kind of auxiliary projection head whose confounding effect this work is explicitly designed to remove. In this sense our approach differs from these foundation models not only in scale but in objective: rather than learning a general-purpose EEG representation, we perform cross-modal knowledge distillation from a frozen CLIP image space into a projection-free EEG latent, aligning EEG directly with the vision–language embedding consumed by downstream generators.

### 2.2. EEG-Driven Generative Frameworks and Baseline Selection

Translating brain activity into visual stimuli has recently become a highly active research area. Initial attempts utilizing generative adversarial networks demonstrated the feasibility of reconstructing visual categories from neural signals [24,25,26,27]. The emergence of diffusion models further accelerated this field, with notable successes first appearing in functional magnetic resonance imaging decoding [28,29].

Inspired by these advancements, state-of-the-art frameworks have adapted diffusion models for EEG signals. Notable examples include DreamDiffusion [30], cascaded diffusion approaches like BrainVis [31], and systems incorporating cross-modal knowledge distillation [32,33]. Crucially, to extract the initial neural features, these modern generative pipelines heavily rely on high-capacity self-supervised backbones, specifically masked autoencoders or time-series specific variants such as Ti-MAE [4]. In particular, DreamDiffusion [30] pre-trains its EEG encoder with temporal masked signal modeling—a masked autoencoder of the same family as the time-series Ti-MAE [4] that we adopt—and then aligns the resulting EEG embedding with the CLIP image embedding space to condition the diffusion model. This CLIP-alignment paradigm is precisely what motivates our design: constraining the encoder to emit a latent that matches the CLIP dimensionality is what allows it to plug into such pipelines, which is why we adopt a CLIP-scale latent rather than an arbitrary bottleneck size.

Because Ti-MAE [4] is representative of the masked-autoencoder EEG encoders on which these contemporary state-of-the-art generative frameworks are built—the temporal masked signal modeling encoder of DreamDiffusion [30] belongs to the same family—it is a task-matched rather than an arbitrary baseline, and we explicitly adopt it as our primary baseline. However, prior studies primarily evaluate these encoders indirectly through end-to-end image synthesis. Such evaluations introduce significant confounding factors, as the final visual quality is heavily dictated by the generative prior of the diffusion model rather than the intrinsic quality of the extracted brainwave features. To reduce the confounding effect of diffusion priors, our work departs from end-to-end generative evaluation. Instead, we directly assess cross-modal semantic alignment through zero-shot retrieval and measure signal reconstruction fidelity, indicating that our lightweight architecture compares favorably with heavily parameterized baselines in extracting structurally faithful latents.

## 3. Method

**Figure 1:**
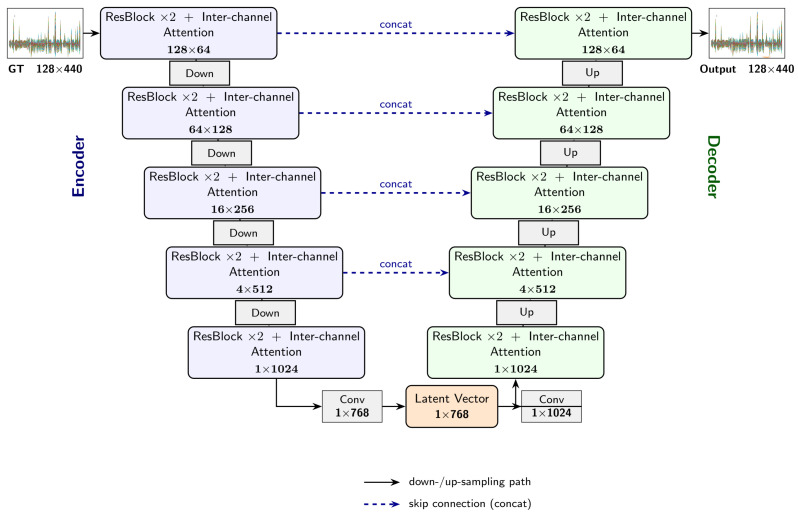
Overview of the proposed U-Net-style autoencoder. The encoder progressively downsamples multi-channel signals using 1D convolutional residual blocks, while attention modules model inter-channel interactions. A fixed-size bottleneck produces a latent vector matching CLIP dimensions without auxiliary projection layers. The decoder mirrors the encoder with skip connections to reconstruct the input signal and preserve fine-grained temporal information. In the input (**top-left**) and output (**top-right**) signal insets, different colors denote the different overlaid EEG channels on a shared time axis and do not encode a quantitative color scale.

Figure 1 summarizes the proposed U-Net-style autoencoder: a convolutional–attention encoder progressively downsamples the multi-channel input to a 768-dimensional bottleneck latent, and a mirrored decoder with skip connections reconstructs the signal. Unlike a broad survey of EEG encoders, this section specifies the concrete architectural choices that constitute our contribution, namely an EEG-tailored convolution–attention encoder and a projection-free 768-dimensional bottleneck. We describe each component in turn below.

### 3.1. Problem Formulation

Let a signal segment be denoted as $X\in R^{C\times T}$, where *C* represents the number of channels, and *T* denotes the number of temporal samples. Our objective is to learn an encoder–decoder mapping $f_{\theta },g_{\phi }$, such that the encoder maps an input signal to a compact latent representation, $z\in R^{D}$, and the decoder reconstructs the original signal from this latent vector. Unlike conventional autoencoders that freely select latent dimensionality, we explicitly constrain *D* to match the dimensionality of CLIP embeddings, specifically D=768. This design enables direct compatibility with generative visual pipelines operating in CLIP-scale latent spaces.

Formally, the encoder and decoder are defined as

(1) $$z=f_{\theta }\left(X\right),\;\hat{X}=g_{\phi }\left(z\right),$$

where $\hat{X}$ denotes the reconstructed signal. The model is trained to minimize reconstruction error under this fixed latent dimension constraint. Because D=768 matches the CLIP embedding dimensionality utilized by many diffusion pipelines, the learned latent vector can be directly injected as a conditioning signal without any post-hoc projection.

### 3.2. Spatiotemporal Motivation

To motivate the encoder design, we view scalp EEG as the forward projection of latent neural sources. At each time step, the measured multi-electrode signal can be expressed as

(2) X(t)=AS(t)+N(t),

where $X\left(t\right)\in R^{C}$ is the observation over *C* electrodes, $A\in R^{C\times K}$ is the (unknown) lead-field mixing matrix, $S\left(t\right)\in R^{K}$ collects the latent neural sources, and N(t) denotes additive noise arising from ocular, muscular, line, and measurement artifacts. Under this view, an encoder that aims to recover a representation reflecting the underlying sources must contend with two coupled structures: the temporal dynamics of each source, and the spatial mixing across electrodes induced by *A*.

This observation directly motivates the two complementary components of our architecture. The one-dimensional convolutional residual blocks operate along the temporal axis to capture local temporal patterns within each channel, whereas the inter-channel attention modules model the cross-electrode dependencies that arise from the spatial mixing. We stress that this generative picture is used only as a design motivation for including both temporal and spatial modeling. It is a linear, idealized view of EEG and is not intended to describe the behavior of the nonlinear, content-dependent attention operator that the network actually learns, nor do we claim optimality of any particular arrangement of these components.

Finally, although the generative view frames the encoder target in terms of the neural sources, our training objective aligns the latent with CLIP image embeddings rather than with the sources directly. These objectives are consistent rather than competing: the recovered source-related structure carries the stimulus information, so projecting it toward a visual-semantic space is compatible with, rather than opposed to, capturing the underlying neural content.

### 3.3. Definition of Projection-Free CLIP-Scale Latent

We define a representation as projection-free if the encoder directly outputs a CLIP-scale latent vector $z\in R^{768}$ without any additional trainable mapping head appended after the encoder, such as a linear mapper, knowledge distillation module, or post hoc alignment network. In our framework, the dimensionality constraint D=768 is satisfied entirely by the architectural design of the encoder bottleneck rather than by a separate projection module, enabling plug-and-play use in CLIP-conditioned generative pipelines.

### 3.4. U-Net-Style Autoencoder Architecture

Masked autoencoders [34] based on vision transformers [35] have become a popular choice for representation learning on complex data. However, directly adopting such architectures for brainwave signals introduces several practical limitations, particularly when the latent dimensionality is constrained to CLIP-scale sizes. To address this issue, we design a U-Net [5]-style autoencoder architecture explicitly tailored to the temporal and spatial characteristics of the input signals. Figure 1 provides an overview of the proposed architecture.

Brainwave data exhibit two key properties: strong temporal dynamics within each channel and pronounced correlations across channels. Based on these observations, the proposed architecture combines convolutional and attention-based components to capture both local and global dependencies. However, as discussed by Park and Kim [36], the strong global connectivity of full-scale transformer models, while expressive, can be detrimental to learning in highly constrained settings. In light of this, we employ hybrid convolution-attention modules that selectively model cross-channel interactions rather than relying on full-scale global self-attention throughout the network.

**Figure 2:**
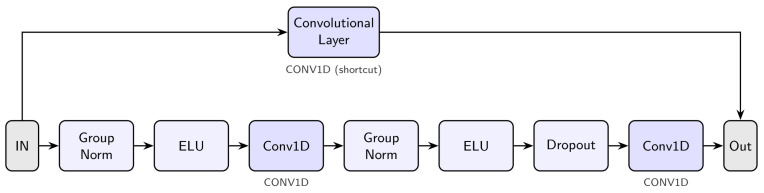
One-dimensional convolutional residual block.

**Figure 3:**
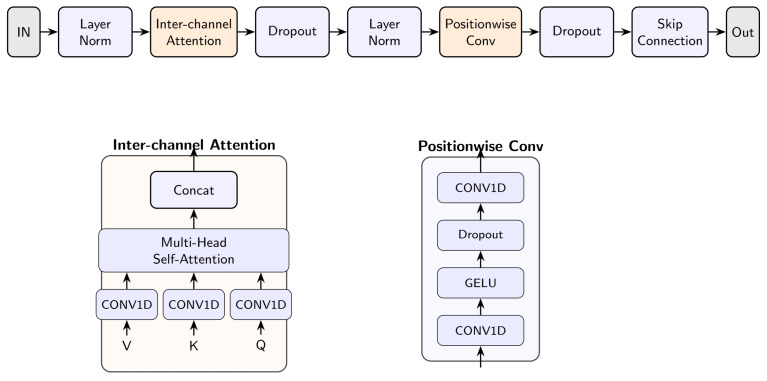
Inter-channel attention module.

The encoder consists of multiple residual blocks constructed from one-dimensional convolutional layers. Each residual block applies one-dimensional convolutions followed by non-linear activation and normalization, enabling the effective modeling of local temporal patterns while maintaining stable gradient flow. To capture global contextual information across channels, we incorporate an inter-channel attention module operating on channel-wise representations. This design complements channel-independent temporal feature extraction by explicitly modeling cross-channel dependencies through attention. Unlike full-scale vision transformers, these modules are designed with limited depth to balance modeling capacity and parameter efficiency. Figure 2 and Figure 3 illustrate the detailed designs of the convolutional block and the attention module, respectively. Concretely, Figure 2 depicts the one-dimensional convolutional residual block: two 1D convolutions, each followed by ELU activation and group normalization, together with an identity shortcut that adds the block input to its output to stabilize gradient flow. Figure 3 depicts the inter-channel attention module: query, key, and value are produced by 1D convolutions over the channel dimension, so that the attention weights model pairwise dependencies between electrodes rather than temporal tokens, and the attended features are subsequently fused back into the convolutional stream.

At the bottleneck (here used in the machine-learning sense of the lowest-dimensional latent layer of the autoencoder, and not in any neurophysiological sense) between the encoder and decoder, the latent representation is compressed into a fixed-size vector of dimension 768. This latent vector directly matches the dimensionality of CLIP embeddings and is produced without any auxiliary projection networks, distinguishing the proposed approach from prior alignment pipelines.

The decoder mirrors the encoder structure and progressively reconstructs the signal from the latent vector. Skip connections between corresponding encoder and decoder stages are employed to preserve fine-grained temporal information and stabilize reconstruction. These connections play a critical role in mitigating information loss caused by aggressive dimensionality reduction at the bottleneck.

### 3.5. Residual and Transformer Blocks

Each convolutional residual block consists of two one-dimensional convolution layers followed by non-linear activation and normalization. We adopt the ELU activation function to better accommodate the negative-valued distributions commonly observed in neurophysiological signals. Group normalization is employed to ensure stable training across varying batch sizes.

The transformer module is designed to model inter-channel dependencies rather than temporal token sequences. Query, key, and value projections are implemented using one-dimensional convolutions, allowing seamless integration with convolutional feature maps. Positional information is incorporated using truncated normalization rather than sinusoidal encoding, reflecting the structured and continuous nature of the input channels. By interleaving convolutional residual blocks with transformer modules, the architecture captures both short-range temporal patterns and long-range channel relationships, which are essential for robust representation learning.

### 3.6. Latent Dimensionality Design

A key design decision in the proposed architecture is the explicit constraint on latent dimensionality. Instead of selecting the bottleneck size solely based on reconstruction performance, we fix the latent vector dimension to match that of CLIP embeddings. This constraint ensures that the learned representations can be directly utilized as conditioning inputs in generative visual models without additional dimensional alignment. Empirically, transformer-based masked autoencoder [34] models exhibit significant performance degradation under similar dimensional constraints, whereas the proposed U-Net [5]-style architecture maintains stable reconstruction quality utilizing substantially fewer parameters.

### 3.7. Architectural vs. Post Hoc Dimension Matching

Matching the CLIP dimensionality can be achieved either by learning an arbitrary embedding and adding an external projection head to map it to the target dimensions, or by explicitly constraining the encoder to produce a 768-dimensional latent directly at the bottleneck. We adopt the latter approach to avoid confounding improvements derived from the capacity of an auxiliary mapper, keeping the conditioning interface identical to standard CLIP embeddings.

We frame this projection-free property as a cleaner evaluation interface rather than as a claim of performance superiority. An auxiliary mapper introduces additional trainable capacity whose contribution would otherwise be entangled with that of the representation itself, so any measured gain could be attributed to the mapper instead of to the encoder. Removing the mapper avoids this confound and yields a more interpretable measurement of the intrinsic latent. We also state the comparison honestly: because the Ti-MAE [4] baseline does not natively emit a 768-dimensional vector, it is equipped with a linear projection, so the Ti-MAE (with linear projection) versus EEGAE (projection-free) comparison already contrasts a projection head against a projection-free design. However, the two systems also differ in their backbone architecture, so the observed advantage is not isolated to the projection-free property alone. Accordingly, we make only the methodological claim that the projection-free interface removes the auxiliary-mapper capacity confound; we do not claim that being projection-free is by itself performance-superior.

### 3.8. Training Objective

We train the autoencoder with a dual objective that couples signal fidelity with semantic alignment. Specifically, we minimize a reconstruction loss $L_{\mathrm{MSE}}$ between the input signal and its reconstruction, while simultaneously optimizing a contrastive alignment loss $L_{\mathrm{con}}$ that pulls the encoded latent *z* toward the corresponding CLIP image embedding and pushes it away from non-matching samples within the batch. This dual-objective setup reflects our target use case in CLIP-conditioned generative pipelines, ensuring the latent vector remains both reconstructive and semantically aligned. The total training objective is defined as the sum of the reconstruction and contrastive alignment losses.

Formally, let $z_{i}$ denote the encoded latent of the *i*-th EEG segment in a mini-batch of size *B*, and let $c_{i}$ be the corresponding CLIP image embedding. Both vectors are $\ell_{2}$-normalized, and $\mathrm{sim}\left(u,v\right)=u^{⊤}v$ denotes cosine similarity. The contrastive alignment loss is a symmetric InfoNCE objective with temperature τ:

(3) $$L_{\mathrm{con}}=-\frac{1}{2B}\sum_{i=1}^{B}\left[\log\frac{\exp(\mathrm{sim}\left(z_{i},c_{i}\right)/\tau )}{\sum_{j=1}^{B}\exp\left(\mathrm{sim}\left(z_{i},c_{j}\right)/\tau \right)}+\log\frac{\exp(\mathrm{sim}\left(z_{i},c_{i}\right)/\tau )}{\sum_{j=1}^{B}\exp\left(\mathrm{sim}\left(z_{j},c_{i}\right)/\tau \right)}\right].$$

For each anchor, $z_{i}$, the matching CLIP embedding $c_{i}$ is the single positive, while the remaining B−1 image embeddings in the same mini-batch serve as in-batch negatives; no external memory bank or hard-negative mining is used. We use a fixed temperature, τ=0.07 (implemented as a logit scale exp(2.6592)≈14.28 that is held constant, i.e., not updated by the optimizer, throughout training) and a batch size of B=64.

## 4. Experiments

The results reported below are our own experiments. We evaluate the proposed encoder directly, without any downstream generative model, to isolate representation quality from the generative prior.

### 4.1. Datasets

We evaluate the proposed autoencoder on two visual perception datasets widely used in neurophysiological decoding and representation learning. The first is the EEGCVPR40 dataset [37,38], which consists of multi-channel recordings collected while subjects viewed visual stimuli. To further rigorously evaluate semantic alignment under highly challenging conditions, we additionally utilize the THINGS EEG2 dataset [39], which provides an extensive and complex set of visual categories. Following standard preprocessing protocols, the signals are segmented into fixed-length temporal windows and standardized on a per-channel basis. For EEGCVPR40, signals are recorded at 1000 Hz; each segment spans 128 electrodes over a 440-sample window (the [20:460] interval of the original 500-sample epochs) and is band-pass filtered to 5–95 Hz. We adopt the standard image-level split, partitioning the data into 7959 training, 1994 validation, and 1987 test segments (approximately 66.6%/16.7%/16.7%), such that no stimulus image appears in more than one split and all 40 categories and all six subjects are represented in every split. No class labels are used during training: the reconstruction objective is self-supervised, whereas the contrastive objective uses paired EEG–image data to align the latent with the corresponding CLIP image embedding. We, therefore, describe the training as label-free with paired cross-modal alignment rather than strictly self-supervised.

For completeness, we summarize the acquisition details of the source datasets as reported in their original studies. EEGCVPR40 was recorded from six subjects using a 128-channel system at 1000 Hz while they viewed images from 40 object categories. THINGS EEG2 was recorded from ten subjects with a 63-channel EEG system while they viewed a large set of natural object images; each epoch spans the −200 to +800 ms interval around stimulus onset (1000 samples), and per-channel standardization is applied identically to EEGCVPR40. Following the dataset’s image-level protocol, the data comprise 272,910 training epochs over 16,540 unique images and 7600 test epochs over 200 held-out concepts, with no stimulus image shared across splits.

### 4.2. Baselines and Evaluation Scope

To assess the effectiveness of the proposed architecture, we adopt Ti-MAE as our primary baseline. Ti-MAE serves as the foundational self-supervised encoder in contemporary state-of-the-art generative frameworks. While prior studies primarily evaluate these encoders indirectly through end-to-end image synthesis, such evaluations introduce confounding factors because the final visual quality is heavily dictated by the generative prior of the diffusion model rather than the intrinsic quality of the extracted features. We evaluate the encoder–decoder representation interface without relying on downstream generative image synthesis, thereby reducing the confounding effect of diffusion priors.

For a fair evaluation under the CLIP-scale interface, all models are required to produce a 768-dimensional latent representation. Because the Ti-MAE baseline does not naturally output latent vectors of this size, we follow common practice and apply a lightweight linear projection to map its native latent space to the target dimension. In contrast, our method satisfies the dimensional constraint by architectural design at the encoder bottleneck, eliminating the need for an auxiliary mapper and avoiding additional capacity that could confound representation quality. We evaluate all methods under identical protocols and report signal reconstruction fidelity, semantic alignment, and parameter counts.

### 4.3. Training Details

All models are trained using a primary reconstruction loss $L_{\mathrm{MSE}}$ coupled with a contrastive alignment loss $L_{\mathrm{con}}$. To ensure a fair comparison, the same optimizer, learning rate, and data splits are applied across the compared methods; their architectures and parameter counts differ by design (Table 1). Training proceeds until convergence based on reconstruction performance on a held-out validation split.

To enable semantic alignment with the visual domain, the contrastive loss optimizes the encoder to maximize the cosine similarity between the generated latent vector and the corresponding ground-truth CLIP image embedding while minimizing similarity with non-matching pairs within the batch. The total training objective is defined as:

(4) $$L=L_{\mathrm{MSE}}+\alpha L_{\mathrm{con}}$$

We set the balancing weight to α=1. To justify this choice, we conduct a sensitivity analysis over α∈{0.1,0.5,1,2,5} on THINGS EEG2 (Table 2). A very small weight (α=0.1) yields the best reconstruction but drives retrieval to near chance, whereas retrieval is broadly stable for α∈[0.5,5] and reconstruction is best near α≈0.5–1. The setting α=1, therefore, lies in a flat region that balances reconstruction fidelity and semantic alignment, which motivates fixing it without further tuning.

For reproducibility, we summarize the model and training configuration. The encoder uses five resolution scales with channel widths {64,128,256,512,1024} and a downsampling factor of four per scale; each residual block contains two convolutional layers, the inter-channel attention module uses four heads, dropout is set to 0.5, and the bottleneck emits the 768-dimensional latent. The models are optimized with AdamW at a fixed learning rate of $1\times 10^{-4}$ (no scheduler), using mixed-precision training on a single GPU. The CLIP-aligned models combine the reconstruction loss with the contrastive objective (α=1) against CLIP ViT-L/14 image embeddings, and the THINGS-EEG2 alignment model uses a batch size of 64.

For full reproducibility, we report the remaining training and hardware details. Models are trained for 31,000 optimization steps with AdamW (learning rate $1\times 10^{-4}$, β=(0.0,0.99)) and no learning-rate schedule, using automatic mixed precision on a single NVIDIA RTX 6000 Ada Generation GPU (48 GB); gradients are clipped to a maximum norm of 1.0 for numerical stability. The contrastive target is the frozen CLIP ViT-L/14 image embedding, precomputed once per image. For the multi-seed statistics reported below, we retrain three random seeds under an identical protocol on a fixed subset of the training epochs held in memory, so that every architectural variant, seed, and α setting is compared under exactly the same conditions.

### 4.4. Evaluation Metrics

Our evaluation protocol is tailored to the specific characteristics of the datasets. For the EEGCVPR40 dataset [37,38], which serves as a standard benchmark in prior studies, we primarily assess basic reconstruction fidelity using Mean Squared Error under the strict CLIP-scale dimensional constraint. We also report the total number of trainable parameters to evaluate architectural efficiency.

To provide a more comprehensive structural and frequency-domain analysis on the highly challenging THINGS EEG2 dataset [39], we expand our evaluation metrics. Alongside Mean Squared Error, we report Mean Absolute Error to quantify point-wise amplitude differences. To evaluate structural and shape preservation after extreme dimensional compression, we utilize the Pearson Correlation Coefficient and Fast Fourier Transform Cosine Similarity.

Furthermore, we assess the cross-modal semantic alignment between the learned representations and visual concepts using the THINGS EEG2 dataset [39]. We perform zero-shot cosine similarity retrieval, measuring Top-1, Top-3, and Top-5 accuracy by calculating the similarity between the generated latents and ground-truth image CLIP embeddings. High retrieval accuracy indicates that the encoder successfully extracts intrinsic semantic content solely from noisy signals. In the retrieval evaluation, local accuracy restricts candidates to the current batch, whereas global accuracy searches across all image embeddings in the entire dataset. Finally, we qualitatively visualize the latent space using t-SNE to verify whether the encoder forms structurally meaningful semantic clusters without relying on generative diffusion priors.

Table 3 reports the reconstruction fidelity, measured by Mean Squared Error, achieved on the EEGCVPR40 dataset under the strict CLIP-scale dimensional constraint.

### 4.5. Qualitative Analysis of Signal Tracking

**Figure 4:**
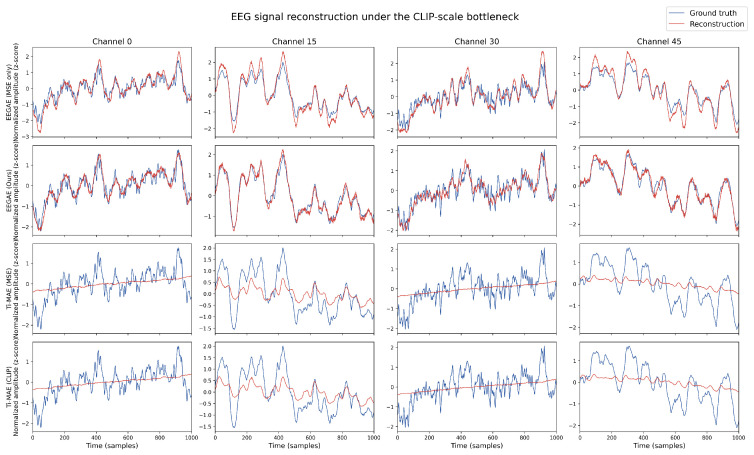
Qualitative comparison of signal reconstruction under the strict CLIP-scale dimensional constraint. Under the imposed CLIP-scale bottleneck, the baseline (**bottom rows**) produces flattened signals with substantially reduced temporal variability, whereas our proposed architecture (**top rows**) more closely tracks high-frequency dynamics.

To further understand the quantitative reconstruction metrics, we qualitatively analyze the reconstructed signals. Figure 4 provides a visual comparison between the ground-truth signals and the outputs of the respective models under the strict 768-dimensional constraint.

In Figure 4, each panel overlays the ground-truth EEG trace with the model reconstruction for a representative channel; the top rows correspond to the proposed EEGAE and the bottom rows to the Ti-MAE baseline under the identical 768-dimensional constraint, and the reader should compare how closely each reconstruction follows the high-frequency peaks and troughs of the ground truth. A notable observation is the collapse-like behavior exhibited by the baseline under this specific configuration. As seen in the bottom rows, Ti-MAE [4] fails to preserve the intricate temporal dynamics, outputting heavily smoothed lines that merely approximate the mean amplitude. This suggests that heavy transformer-based architectures tend to lose high-frequency structural information during aggressive dimensionality reduction. Conversely, the proposed U-Net-style encoder more closely tracks the fine-grained peaks and valleys of the original brainwaves. This indicates that the integration of one-dimensional convolutional residual blocks and skip connections helps preserve critical temporal structures even at the extreme bottleneck.

Table 4 reports zero-shot retrieval accuracy on EEGCVPR40 for both local (within-batch) and global (whole-dataset) candidate pools, showing that retrieval remains well above chance in both regimes.

Table 5 compares comprehensive reconstruction fidelity on the more challenging THINGS EEG2 dataset across time- and frequency-domain metrics.

Table 6 reports zero-shot Top-*k* retrieval on THINGS EEG2, where the random-chance level is approximately 0.005.

### 4.6. Latent Space Clustering and Semantic Alignment

While reconstruction quality measures low-level signal fidelity, it does not guarantee that the model understands the semantic content of the visual stimuli. As reported in Table 6, our model retrieves above the random-chance level on the highly complex THINGS EEG2 dataset [39], where the baseline remains at chance. We emphasize that the absolute accuracy is modest: the Top-1 accuracy of 0.0185 is roughly 3.7 times the random-chance level of approximately 1/200=0.005 (the retrieval is performed against 200 candidate images), which indicates a consistent above-chance signal while leaving substantial room for improvement. This modest absolute value is expected rather than anomalous: THINGS EEG2 is a deliberately hard benchmark: retrieval is single-trial (no trial averaging), the gallery spans 200 fine-grained object concepts, and single-trial EEG has an intrinsically low signal-to-noise ratio. Under these conditions even a consistently above-chance latent is informative, and the gap to the baseline—which stays at chance—is the meaningful signal. We expect that scaling to larger paired EEG–image datasets and trial averaging would further raise this alignment. Qualitative examples of the EEGCVPR40 retrieval (Table 4), covering both success and failure cases, are provided in Appendix A, which also includes an illustrative EEG-to-image generation proof of concept.

**Figure 5:**
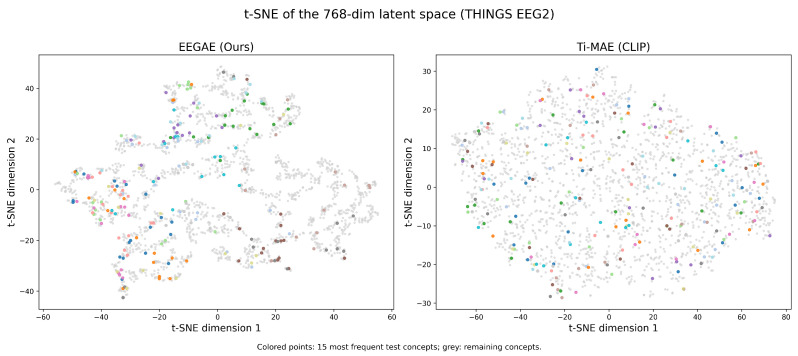
t-SNE visualization of the learned 768-dimensional latent spaces on the THINGS EEG2 dataset [39]. Unlike the baseline (**right**), whose latent space shows little visible category structure (consistent with near-chance retrieval), our proposed model (**left**) exhibits emerging, partially separated semantic structure rather than a single undifferentiated cloud. Colored points mark the fifteen most frequent test concepts and remaining concepts are shown in grey; the horizontal and vertical axes are the two dimensionless t-SNE embedding coordinates and carry no physical unit, so only the relative arrangement of points is meaningful.

This semantic alignment is further illustrated through qualitative latent space visualizations. Figure 5 presents the t-SNE projections of the 768-dimensional latent vectors. The baseline latent space shows little visible category structure, consistent with its near-chance retrieval performance. In contrast, our proposed architecture exhibits emerging, partially separated structure rather than a single undifferentiated cloud. This qualitative trend is consistent with the above-chance retrieval results and suggests that the projection-free design begins to capture semantic structure directly from noisy signals without relying on downstream generative priors; we note that the separation is partial and leaves substantial room for improvement. We treat this visualization as qualitative evidence only; quantitative cluster-quality metrics, such as the silhouette score or *k*-nearest-neighbor category consistency, are left to future work.

### 4.7. Ablation Studies

#### 4.7.1. Impact of Contrastive Alignment

We first evaluate the necessity of the dual-objective training strategy by isolating the impact of the contrastive alignment loss. We compare a variant of our model trained exclusively with Mean Squared Error against the fully equipped model trained with both reconstruction and contrastive objectives. The results are detailed in Table 5 and Table 6.

When trained solely on reconstruction, the encoder closely preserves the physical signal structure, achieving the highest Pearson Correlation Coefficient (0.940, Table 5). However, this configuration completely fails to align with the visual domain, yielding a Top-1 retrieval accuracy of exactly 0.0050, which equates to random chance. Incorporating the contrastive loss resolves this semantic gap, elevating the Top-1 retrieval accuracy to 0.0185 and the Top-5 accuracy to 0.0901, at only a small cost in reconstruction fidelity (PCC 0.940 to 0.902). These findings indicate that the dual-objective formulation is essential: the contrastive term is what bridges the modality gap to enable semantic alignment, while the reconstruction term preserves signal fidelity.

#### 4.7.2. Impact of Architectural Components

We further ablate the individual architectural components by removing, in turn, the inter-channel attention module, the residual blocks, and the U-Net skip connections, keeping all else fixed and averaging over three seeds (Table 7). Removing the residual blocks or the skip connections is catastrophic: the reconstruction error increases roughly three- to fourfold and retrieval collapses to chance, confirming that both components are essential to the design. Removing the inter-channel attention module, by contrast, does not degrade performance in this setting—and in fact slightly improves both reconstruction and retrieval while reducing seed variance. We, therefore, do not claim that the inter-channel attention module improves reconstruction or retrieval; we retain it as an optional component of the architecture and leave to future work the question of whether cross-channel attention benefits downstream generative conditioning, where cross-channel context may play a different role than in the reconstruction and retrieval tasks measured here.

## 5. Discussion

Self-supervised encoders that serve as conditioning backbones for generative visual models are typically inherited from large masked-autoencoder and transformer designs developed for generic vision or time-series data. When such encoders are repurposed for EEG-to-image generation under a CLIP-scale interface, three difficulties can emerge. First, the encoder does not natively emit a CLIP-dimensional vector and, therefore, requires an auxiliary projection head, which adds trainable capacity and confounds the evaluation of the representation itself. Second, forcing a heavy transformer bottleneck down to the small CLIP dimension induces representation collapse, flattening the reconstructed signal and degrading the latent space into an unstructured cloud. Third, generic backbones are agnostic to the specific structure of neurophysiological signals. Our results indicate that overcoming these difficulties does not require a larger model, but rather an architecture aligned with the characteristics of the signal.

The spatiotemporal view of Section 3.2 frames this alignment. EEG is well described as the spatial mixing of temporally structured neural sources: each channel carries strong within-channel temporal dynamics, while volume conduction induces pronounced cross-channel correlations. A generic vision-transformer masked autoencoder does not exploit this dual structure. In contrast, our encoder pairs one-dimensional convolutional residual blocks, which model local temporal patterns, with inter-channel attention modules intended to model cross-electrode dependencies, and links the encoder and decoder through skip connections that protect fine-grained temporal information from the aggressive bottleneck. The component ablation (Table 7) shows that the residual blocks and the skip connections are the decisive elements: removing either collapses both reconstruction and retrieval. The inter-channel attention module, however, is not necessary for these metrics in our setting, so we retain it only as an optional component and do not attribute the observed performance to it. This signal-aligned design is what allows the model to track high-frequency dynamics without collapsing (Figure 4) while using substantially fewer parameters than the masked-autoencoder baseline (Table 1).

The projection-free bottleneck addresses the first difficulty directly. By emitting the 768-dimensional latent intrinsically, the model removes the auxiliary mapper and the capacity confound it introduces, yielding a cleaner measurement of the intrinsic representation. Consistent with the framing of Section 3.7, we regard this as a methodological advantage that isolates the contribution of the representation, rather than as a claim that the projection-free property is performance-superior in isolation.

It is also important to distinguish what each evaluation measures. The reconstruction metrics (Table 3 and Table 5) reflect the full encoder–decoder, including the U-Net skip connections, and therefore characterize the system’s signal-reproduction capacity rather than the standalone capacity of the bottleneck latent. The semantic evaluations, in contrast, are computed directly on the 768-dimensional bottleneck latent: zero-shot retrieval (Table 6) and the t-SNE visualization (Figure 5) are based on the cosine similarity between this latent and CLIP image embeddings and are thus not affected by the skip connections. Reconstruction and semantic alignment, therefore, probe complementary and largely independent aspects of the model—the skip connections stabilize reconstruction, whereas the above-chance retrieval reflects information carried by the bottleneck latent itself.

Taken together, the reconstruction results (Table 3 and Table 5) show that the EEG-tailored design preserves signal fidelity that the heavy baseline loses, and the retrieval results (Table 6) show emerging cross-modal alignment that is several times above chance, whereas the baseline remains at the chance level. At the same time, the absolute retrieval accuracy is modest, which we interpret as evidence that the encoder captures partial rather than complete semantic structure; closing this gap, for example by coupling the encoder with stronger downstream generative priors or larger paired datasets, is left to future work. We also note that the contrastive objective is essential to bridging the modality gap: reconstruction alone preserves signal structure but does not, by itself, align it with the visual-semantic space. Overall, these observations support the view that adapting the self-supervised backbone to the spatiotemporal characteristics of EEG, rather than scaling a generic encoder, is an effective route to compact and semantically aligned representations for brain-driven generative pipelines.

We further situate our results relative to prior EEG representation learning rather than reporting them in isolation. Because absolute retrieval and reconstruction numbers are strongly dependent on the dataset, montage, and preprocessing, a direct numerical comparison against results reported on other benchmarks would be confounded; we therefore compare against a matched masked-transformer baseline (Ti-MAE) under an identical protocol and with multi-seed statistics. Under this controlled interface, our projection-free encoder attains a Top-1 retrieval of 0.0185 on THINGS EEG2—significantly above the baseline, which remains at chance—while using substantially fewer parameters and no auxiliary projection head. We emphasize relative trends measured under this identical interface rather than cross-study absolute values.

### Limitations and Future Work

We delineate the scope of our claims through the following limitations. First, the evaluation uses a single primary baseline, Ti-MAE, selected because it is the de facto backbone in comparable EEG-to-image pipelines; a broader empirical comparison against general-purpose EEG foundation models (e.g., LaBraM, EEGPT, CBraMod, EEGFormer) would further establish the advantage of the proposed design. Because those models are trained for montage- and task-agnostic transfer and do not natively emit a CLIP-scale latent, a fair comparison would require appending the very projection head this work removes; we, therefore, treat a direct empirical comparison as future work and rely here on the projection-free positioning argument (Section 2) together with the Ti-MAE baseline, which belongs to the same masked-transformer family and remains at retrieval chance under our protocol. Second, to bound compute, the multi-seed and ablation experiments are trained on a fixed subset of the training epochs held in memory; this controlled protocol reproduces the reconstruction fidelity of the full-data model and preserves all relative comparisons, but the absolute retrieval accuracy under it is modestly lower than the full-data result, so absolute retrieval numbers should be read together with this note. Third, because the U-Net skip connections route information around the 768-dimensional bottleneck, the reconstruction metrics characterize the full encoder–decoder rather than the standalone capacity of the CLIP-scale latent; measuring that capacity directly requires a latent-only decoder with the skip connections disabled at evaluation, which we treat as future work and which tempers any interpretation of reconstruction fidelity as a direct measure of latent richness. Fourth, the cross-modal alignment is modest in absolute terms—Top-1 retrieval is several times above chance but remains low—which we attribute to the difficulty of THINGS EEG2 and the low signal-to-noise ratio of single-trial EEG; scaling to larger paired EEG–image datasets is a promising route to stronger alignment. Regarding subject-independent generalization, we additionally evaluated a subject-disjoint split on THINGS EEG2 (training on a subset of subjects and testing on entirely held-out subjects): reconstruction and retrieval showed no substantial degradation relative to the subject-mixed split (MSE 0.098 vs. 0.095, PCC 0.884 vs. 0.890, Top-1 retrieval in the same range), indicating that the latent generalizes to unseen subjects rather than memorizing subject identity. Finally, the EEGCVPR40 evaluation uses a subject-mixed image-level split; although this split prevents stimulus-image leakage across partitions and an identical split is applied to every compared model, EEGCVPR40 is also subject to the block-design concern raised by Li et al. [40], so absolute EEGCVPR40 numbers should be interpreted with this caveat in mind while the relative comparison between models remains fair.

## 6. Conclusions

In this paper, we proposed a lightweight and projection-free autoencoder based on a U-Net-style architecture, explicitly designed to learn efficient and semantically aligned representations from neurophysiological signals. Unlike conventional masked autoencoder approaches that rely on heavily parameterized bottlenecks and auxiliary alignment networks, our model directly outputs compact, CLIP-scale latent vectors that are trained to align with the CLIP embedding space, with the alignment emerging rather than complete. By combining one-dimensional convolutional residual blocks with inter-channel attention modules, the architecture effectively captures both local temporal dynamics and global spatial correlations while preventing information loss through skip connections.

In summary, the contributions of this work are as follows. First, we propose a projection-free autoencoder that directly produces CLIP-scale latent representations without auxiliary alignment networks. Second, we present a U-Net-style architecture that mitigates collapse-like degradation under strict dimensional constraints by balancing temporal modeling, inter-channel dependency capture, and parameter efficiency. Last, we empirically demonstrate that our approach achieves superior signal tracking and shows emerging, partially structured semantic organization, suggesting a compact alternative to heavily parameterized baseline models for visual perception tasks.

Extensive evaluations on the EEGCVPR40 and the highly challenging THINGS EEG2 datasets [39] demonstrated that our approach achieves superior signal reconstruction fidelity utilizing substantially fewer parameters than the baseline models. Crucially, under the strict CLIP-scale bottleneck, the Ti-MAE [4] baseline exhibited collapse-like behavior, failing to preserve signal dynamics. In contrast, our proposed model better preserved high-frequency temporal structures across both time and frequency domains.

Furthermore, zero-shot retrieval tasks and latent space visualizations indicated that our projection-free design captures emerging semantic structure directly from noisy signals, performing several times above the random-chance level to which the baseline degenerated, while the absolute retrieval accuracy leaves substantial room for improvement. By evaluating the encoder without relying on downstream generative priors, we provide evidence for the efficacy of our structural design. We believe this efficient and compact encoder provides a practical foundation for future research in multimodal representation learning and interactive brain-driven visual applications.

## Figures and Tables

**Table 1:** Comparison of trainable parameters between the baseline and the proposed model across different datasets.

**Model**	Parameters (M)	
	**EEGCVPR40**	**THINGS EEG2**
Ti-MAE [4]	98.8	90
EEGAE (Ours)	56.6	49

**Table 2:** Sensitivity of THINGS EEG2 performance to the loss balancing weight α (single seed, revision protocol). Reconstruction is best at small α, whereas retrieval requires α≳0.5; α=1 lies in a flat region that balances both. Arrows denote the preferred direction (↑: higher is better; ↓: lower is better), and the best value in each column is shown in bold.

α	MSE ↓	PCC ↑	Top-1 ↑	Top-5 ↑
0.1	0.154	**0.930**	0.007	0.028
0.5	0.083	0.912	**0.021**	**0.094**
1.0	**0.082**	0.898	0.014	0.082
2.0	0.083	0.892	0.019	0.089
5.0	0.092	0.885	0.020	0.082

**Table 3:** Reconstruction performance on the EEGCVPR40 dataset [37,38]. The arrow denotes the preferred direction (↓: lower is better).

Model	MSE ↓
Ti-MAE [4]	0.0674
EEGAE (Ours)	0.0115

**Table 4:** Quantitative results of cosine similarity retrieval task. We report Top-k accuracy for both local (within batch) and global (entire dataset) search scopes on the EEGCVPR40 dataset [37,38]. Arrows denote the preferred direction (↑: higher is better).

Dataset	Scope	Top-1 ↑	Top-3 ↑	Top-5 ↑
Validation	Local	0.35	0.60	0.78
	Global	0.26	0.51	0.66
Test	Local	0.37	0.62	0.79
	Global	0.24	0.52	0.69

**Table 5:** Comprehensive reconstruction fidelity on the highly challenging THINGS EEG2 dataset [39]. Values are mean ± standard deviation over three random seeds; best per column in bold. Arrows denote the preferred direction (↑: higher is better; ↓: lower is better).

Model	MSE ↓	MAE ↓	PCC ↑	FFT-COS ↑
Ti-MAE [4] (MSE)	0.387±0.025	0.490±0.018	0.406±0.035	0.738±0.030
Ti-MAE [4] (CLIP)	0.402±0.003	0.503±0.002	0.411±0.002	0.770±0.006
EEGAE (MSE)	0.097±0.013	0.241±0.018	0.940±0.001	0.961±0.001
EEGAE (CLIP)	0.096±0.024	0.234±0.033	0.902±0.005	0.942±0.003

**Table 6:** Zero-shot Top-K retrieval accuracy on the THINGS EEG2 dataset [39] (Random chance ≈ 0.005). Values are mean ± standard deviation over three random seeds; best per column in bold. Arrows denote the preferred direction (↑: higher is better).

Model	Top-1 ↑	Top-3 ↑	Top-5 ↑
Ti-MAE (MSE)	0.0050±0.0003	0.0151±0.0001	0.0250±0.0001
Ti-MAE (CLIP)	0.0050±0.0001	0.0150±0.0000	0.0249±0.0002
EEGAE (MSE)	0.0050±0.0000	0.0150±0.0000	0.0250±0.0001
EEGAE (CLIP)	0.0185±0.0060	0.0515±0.0147	0.0901±0.0162

**Table 7:** Component-wise ablation on THINGS EEG2 (EEGAE with CLIP alignment). Each variant removes exactly one component; mean ± standard deviation over three seeds. Removing the residual blocks or skip connections collapses both reconstruction and retrieval. Arrows denote the preferred direction (↑: higher is better; ↓: lower is better).

Variant	Params (M)	MSE ↓	PCC ↑	Top-1 ↑	Top-5 ↑
Full model	49.4	0.096±0.024	0.902±0.005	0.019±0.006	0.090±0.016
w/o inter-channel attention	38.2	0.074±0.002	0.911±0.001	0.043±0.007	0.176±0.023
w/o residual blocks	43.1	0.288±0.016	0.589±0.018	0.005±0.000	0.025±0.000
w/o skip connections	51.4	0.402±0.003	0.408±0.007	0.010±0.004	0.056±0.027

**Figure A1:**
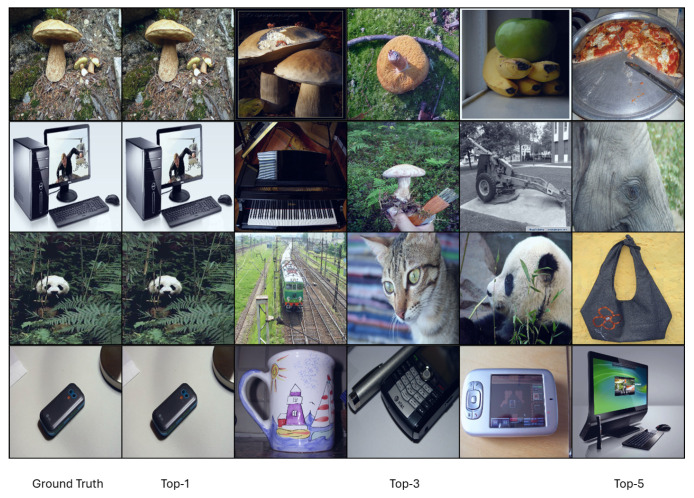
Qualitative zero-shot retrieval on EEGCVPR40 (success cases). For each EEG query, the ground-truth stimulus image and the Top-1 to Top-5 retrieved images are shown.

**Figure A2:**
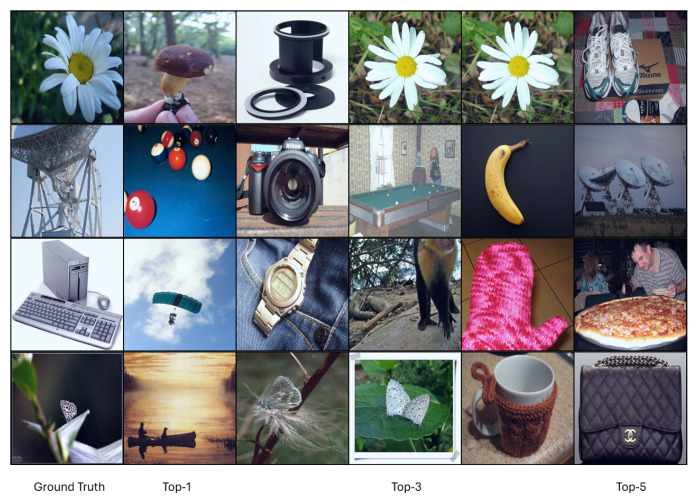
Qualitative zero-shot retrieval on EEGCVPR40 (failure cases), illustrating queries for which the top-ranked retrieved images belong to categories other than the ground truth.

**Figure A3:**
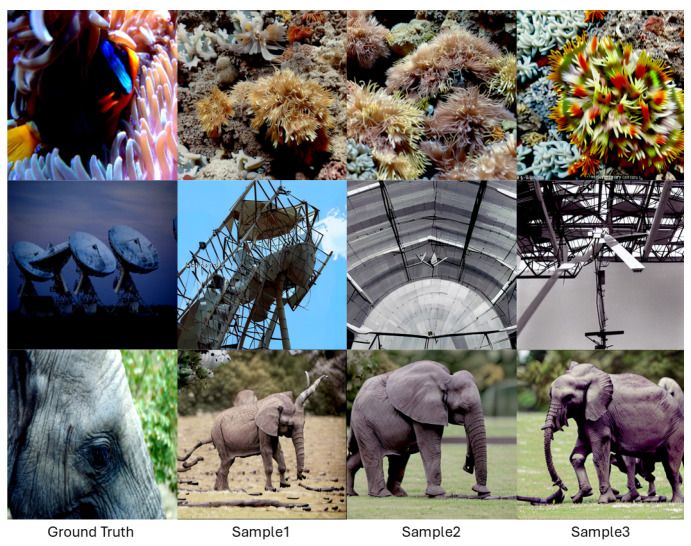
Illustrative EEG-to-image generation on EEGCVPR40. Each row shows the ground-truth stimulus and multiple stochastic samples produced by a frozen Stable Diffusion v1.5 model conditioned on the EEGAE latent through an IP-Adapter. This proof of concept is qualitative; the visual quality reflects the diffusion prior rather than the encoder alone.

## Data Availability

The EEGCVPR40 and THINGS EEG2 datasets used in this study are publicly available from their respective original publications [37,38,39]. The EEGCVPR40 dataset is available at https://github.com/perceivelab/eeg_visual_classification (accessed on 15 July 2026).

## Abbreviations

The following abbreviations are used in this manuscript:

EEG	Electroencephalography
CLIP	Contrastive Language-Image Pre-training
MAE	Masked Autoencoder
MSE	Mean Squared Error
MAE (metric)	Mean Absolute Error
PCC	Pearson Correlation Coefficient
FFT	Fast Fourier Transform
t-SNE	t-Distributed Stochastic Neighbor Embedding
GAN	Generative Adversarial Network
fMRI	Functional Magnetic Resonance Imaging
ELU	Exponential Linear Unit
GELU	Gaussian Error Linear Unit

## Appendix A. Qualitative Examples

To complement the quantitative retrieval scores in Table 4, we provide qualitative zero-shot retrieval results on EEGCVPR40. For each EEG query, we show the ground-truth stimulus image alongside the Top-1 to Top-5 retrieved images, ranked by cosine similarity between the encoded latent and the candidate image CLIP embeddings. Figure A1 presents representative success cases, in which the Top-1 retrieved image matches the ground-truth category, whereas Figure A2 presents failure cases, in which the closest retrieved images belong to different categories. These examples are illustrative and are consistent with the modest absolute retrieval accuracy reported in the main text; they are computed directly on the bottleneck latent and, therefore, do not depend on any downstream generative model.

**EEG-to-image generation.** As a further illustrative proof of concept, we pair the EEGAE encoder with a frozen Stable Diffusion v1.5 model (frozen VAE and U-Net, DDIM sampling): the 768-dimensional EEG latent is mapped by a lightweight IP-Adapter and used as additional cross-attention conditioning for the U-Net. The IP-Adapter is used only after the encoder for visualization and is excluded from all quantitative reconstruction and retrieval evaluations; no adapter-based result supports the projection-free claim in the main experiments. This is a qualitative demonstration of downstream applicability rather than a contribution of this paper—the visual quality is largely governed by the frozen diffusion prior, which is precisely why our quantitative evaluation isolates the encoder from such priors. Figure A3 shows, for several EEG queries, the ground-truth stimulus alongside multiple stochastic samples.
